# Gene therapy with an improved doxycycline-regulated plasmid encoding a tumour necrosis factor-alpha inhibitor in experimental arthritis

**DOI:** 10.1186/ar2113

**Published:** 2007-01-25

**Authors:** David Gould, Nasim Yousaf, Rewas Fatah, Maria Cristina Subang, Yuti Chernajovsky

**Affiliations:** 1 Bone and Joint Research Unit, Barts and The London, Queen Mary's School of Medicine and Dentistry, Charterhouse Square, University of London, London, EC1M 6BQ, UK

## Abstract

Inhibition of tumour necrosis factor (TNF)-alpha with biological molecules has proven an effective treatment for rheumatoid arthritis, achieving a 20% improvement in American College of Rheumatology score in up to 65% of patients. The main drawback to these and many other biological treatments has been their expense, which has precluded their widespread application. Biological molecules could alternatively be delivered by gene therapy as the encoding DNA. We have developed novel plasmid vectors termed pGTLMIK and pGTTMIK, from which luciferase and a dimeric TNF receptor II (dTNFR) are respectively expressed in a doxycycline (Dox)-regulated manner. Regulated expression of luciferase from the self-contained plasmid pGTLMIK was examined *in vitro *in a variety of cell lines and *in vivo *following intramuscular delivery with electroporation in DBA/1 mice. Dox-regulated expression of luciferase from pGTLMIK of approximately 1,000-fold was demonstrated *in vitro*, and efficient regulation was observed *in vivo*. The vector pGTTMIK encoding dTNFR was delivered by the same route with and without administration of Dox to mice with collagen-induced arthritis. When pGTTMIK was delivered after the onset of arthritis, progression of the disease in terms of both paw thickness and clinical score was inhibited when Dox was also administered. Vectors with similar regulation characteristics may be suitable for clinical application.

## Introduction

Tumour necrosis factor-alpha (TNF-α) inhibitors, either antibodies to TNF-α (infliximab, adalimumab) or TNF receptors (TNFRs) fused to an immunoglobulin G-Fc backbone (etanercept), used in combination with methotrexate are the most effective disease-modifying agents for rheumatoid arthritis (RA) in terms of improvement in symptoms, quality of life, and prevention of structural damage. Twenty percent improvements in American College of Rheumatology (ACR) score are achieved in approximately 65% of patients [1]. However, the cost of treatment is high (approximately €10,000 per year), which limits their worldwide use.

The success of anti-TNF-α biologicals in the treatment of RA provides a well-characterised target to incorporate in a gene therapy strategy for the treatment of the disease. Due to the fact that RA is a chronic non-fatal disease, an absolute requirement for any gene therapy treatment is that it be completely safe and ideally have long-term effects. Plasmid DNA, unlike a virus, is devoid of protein components and is therefore non-immunogenic. This quality and its inability to integrate into the genome have established plasmid DNA as a safe gene-delivery vector. However, the absence of an innate mechanism to enter cells has also limited the widespread application of plasmid DNA in gene therapy. But the ability of plasmid DNA to efficiently transfect skeletal muscle, originally reported by Wolff and colleagues [2], has enabled use of plasmid in gene therapy clinical trials [3,4] and in experimental models. When combined with electroporation, the transfection efficiency of skeletal muscle is further enhanced by 100-fold [5], and reporter gene expression is demonstrated in excess of 250 days. Therefore, plasmid DNA can be delivered efficiently *in vivo*, achieving long-term expression, and because it is not immunogenic can potentially be re-administered.

Despite being a chronic disease, RA can go into periods of remission [6] and so the use of regulated promoters will enable the expression of therapeutic molecules to be reduced or switched 'off' during these phases of the disease. Regulated expression will also enhance the safety of the system should adverse effects occur or a second pathology develop. Several pharmacologically regulated systems of gene expression have been developed, including the tetracycline system, which uses the bacterial components of tetracycline resistance in a synthetic system that functions efficiently in eukaryotic cells [7,8]. These original components have been used in a variety of *in vitro *systems, *in vivo *for gene therapy applications, and in transgenic organisms. They have also been combined in self-contained vectors that facilitate their application in gene therapy as only a single plasmid needs to be delivered to cells. Regulated expression with the original tetracycline system is optimal in stably transfected cells, whereas expression in transiently transfected cells is approximately 50- to 100-fold [9-11]. The high basal activity of the tetracycline-responsive promoter (P*tet*) compromised the function of the system in these situations. Improved components have since been developed, including the *tet*R-KRAB (tetracycline repressor-Kruppel-associated box), which binds the P*tet *and reduces basal activity in the absence of antibiotic [12]. An improved transactivator, rtTA2S-M2, was generated that has greater stability than rtTA (reverse tetracycline transactivator) and is also responsive to a tenth of the concentration of doxycycline (Dox) [13]. These improved components can be used in tandem to give more efficient gene regulation *in vitro *and *in vivo *and have also been incorporated into self-contained vectors that function efficiently *in vitro *and *in vivo *[14-16].

In this study, we have constructed a self-contained plasmid vector that incorporates the improved components for tetracycline-regulated gene expression and displays more efficient gene regulation in a variety of transfected cells when compared to a self-contained vector with the original tetracycline-regulated components. Efficient regulated gene expression is also observed *in vivo*, where the vector is maintained long-term. When the TNF-α inhibitor dimeric TNF receptor II (dTNFR) consisting of two extracellular domains of hTNFRII linked by a flexible serine-glycine linker [17] was encoded from the vector, efficient regulation is observed *in vitro *and progression of arthritis is inhibited in an experimental model.

## Materials and methods

### DNA and cells

Plasmid DNA was propagated in DH5-α *Escherichia coli *and was purified using a standard Plasmid Mega Kit (Qiagen Ltd., Crawley, West Sussex, UK); when DNA was prepared for *in vivo *application, the EndoFree™ Plasmid Mega Kit (Qiagen Ltd.) was used. Human skin epithelial cell line A431 (European Collection of Cell Cultures [ECACC] no. 85090402), mouse myoblast cell line C2C12 (ECACC no. 91031101), human embryonic kidney epithelial cell line 293T, mouse DBA/1 embryonic fibroblasts with the temperature-sensitive large T antigen (DTF) [18], and monkey kidney fibroblast Cos7 (ECACC no. 87021302) were grown at 10% CO_2 _in Dulbecco's modified Eagle's medium (BioWhittaker, now part of Cambrex Bio Science Verviers S.p.r.l., Verviers, Belgium) supplemented with 10% foetal bovine serum (Gibco-BRL, now part of Invitrogen Corporation, Carlsbad, CA, USA), glutamine (2 mM) (Cambrex Bio Science Verviers S.p.r.l.), penicillin (100 U/ml) (Cambrex Bio Science Verviers S.p.r.l.), and streptomycin (100 μg/ml) (Cambrex Bio Science Verviers S.p.r.l.). Unless stated otherwise, reagents were purchased from Sigma-Aldrich (St. Louis, MO, USA).

### Plasmid construction

**Figure 1:**
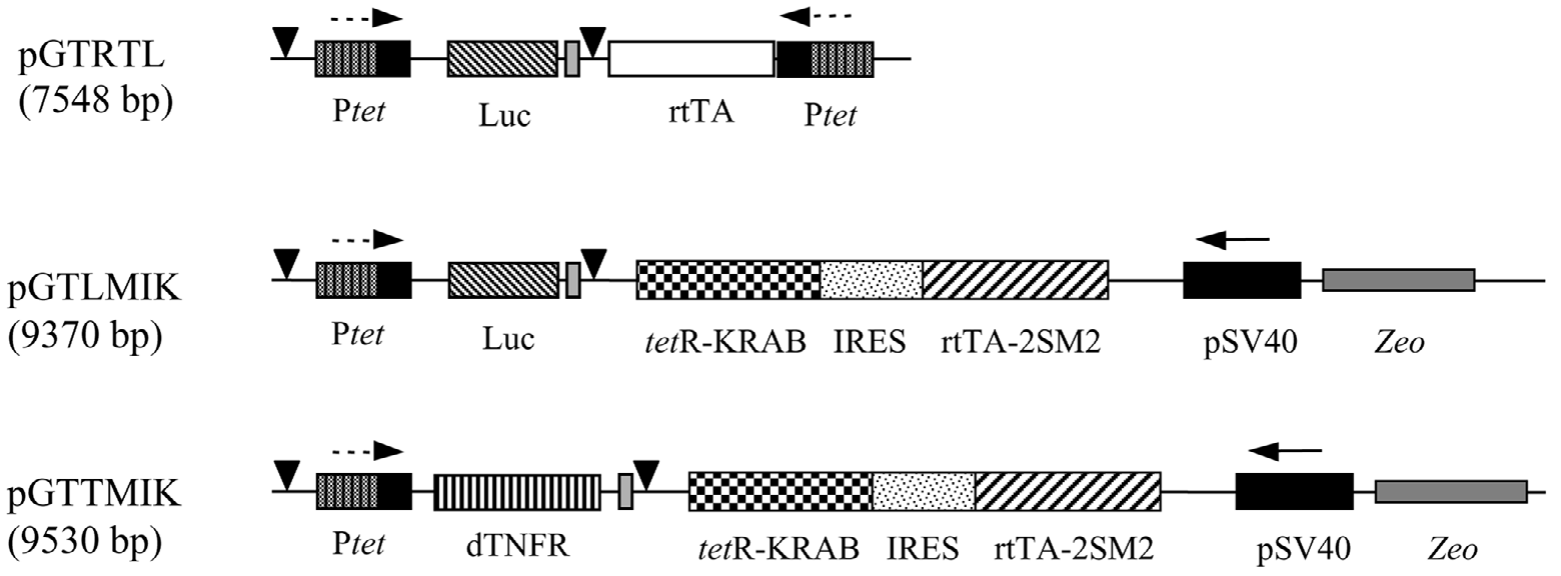
Schematic representation of the vectors used in this study. All vectors are plasmid DNA constructed on the backbone of pGL2-Basic. Promoters are either the constitutive SV40 (pSV40) or tetracycline-responsive (P*tet*). Encoded transgenes are the reverse tetracycline transactivator (rtTA), the improved version (rtTA2S-M2), the tetracycline targeted repressor-Kruppel-associated box (*tet*R-KRAB), firefly luciferase (Luc), or the dimeric tumour necrosis factor receptor II (dTNFR). Other DNA elements are the internal ribosome entry site (IRES) derived from encephalomyocarditis virus, the SV40 polyadenylation signal (black triangle), and the spacer sequence of the non-coded zeocin (*Zeo*) resistance gene.

The plasmids pGTL, pGTRTL [11], pGTRTT, pcLuc+ [19], and pMIK/ZeoSV2 [20] have been described previously. To facilitate cloning, the sense and anti-sense oligonucleotides of sequences GATCTTAAGCCATACCCGGGATCGGGATCCGACTTGG and TCGACCAAGTCGGATCCCGATCCCGGGTATGGCTTAA, respectively, containing restriction sites for BamHI, SmaI, and AflII, were annealed and cloned into the plasmid pGL2-Basic (Promega Corporation, Madison, WI, USA) restricted with BamHI and SalI, producing the plasmid pGLinker. The MIK cassette containing the improved tetracycline transactivator (rtTA2S-M2) and the repressor *tet*R-KRAB from an SV40 promoter along with the upstream *zeo *gene were removed from pMIK/ZeoSV2 by restriction with MscI and AflII and cloned into pGLinker cut with PshA1 and AflII, producing the vector pGMIK. The self-contained plasmid pGTLMIK was then generated by removing P*tet*-Luc from pGTL with XhoI-EcoRV and was cloned into pGMIK restricted with the same enzymes. Finally, pGTTMIK encoding dTNFR was constructed by removing P*tet*-Luc from pGTLMIK with the enzymes MluI-EcoNI and by replacing it with P*tet*-dTNFR removed from pGTRTT with the same enzymes. The principal plasmids used in this study are depicted schematically in Figure 1.

### Cell transfection

Transfections of 293T, A431, DTF, and Cos7 cells were performed using the calcium phosphate precipitation method as described previously [11]. Cells were plated on 12-well plates at a density of 0.2 × 10^6 ^per well and were transfected the next day with 2 μg of DNA. Cells were subjected to an osmotic shock on the second day, after which fresh media was added with or without Dox at a concentration of 1 μg/ml. Levels of luciferase in transfected cells were determined in cell lysates, or dTNFR was measured in supernatants by enzyme-linked immunosorbent assay (ELISA) (see below).

The myoblast cell line C2C12 was transfected by nucleofection. These cells were prepared at 1 × 10^6 ^per 100 μl and were transfected with 2 μg of GTLMIK by means of the amaxa system (amaxa GmbH, Cologne, Germany) and buffers in kit V with program B-032. Cells were then plated in 12-well plates, and fresh medium was added up to 1 ml in each well. Where necessary, Dox (1 μg/ml) was added to the well and luciferase activity was determined 24 and 48 hours later.

### Luciferase assay

Luciferase activity was determined using the luciferase assay system (Promega Corporation). Briefly, cells were washed twice with phosphate-buffered saline (PBS) and were then lysed with passive lysis buffer at room temperature for 15 minutes. Cells were then scraped off plates and transferred to an Eppendorf tube. For analysis, samples were centrifuged for 5 minutes at 13,000 rpm. A 10-μl aliquot of the sample was automatically mixed with 50 μl of luciferase assay substrate, and light emission was measured using an MLX Microtiter^® ^Plate Luminometer (Dynex Technologies, Chantilly, VA, USA). Protein concentrations of cell lysates were determined using the Bradford protein assay (Bio-Rad Laboratories, Inc., Hercules, CA, USA), and values of luciferase activity were expressed as relative light units (RLU) per microgram of protein.

### *In vivo *plasmid DNA delivery

Mice were treated according to approved Home Office and institutional guidelines. Naïve or arthritic DBA/1 mice were given an intraperitoneal (i.p.) injection of the muscle relaxant Hypnorm™ (Janssen Animal Health, Janssen Pharmaceuticals, Antwerp, Belgium) and were anaesthetised with halothane (Concord Pharmaceuticals Ltd, Horsham, West Sussex, UK) using Boyle's apparatus (British Oxygen Company, now part of Linde AG, Wiesbaden, Germany). The fur covering the right quadricep was shaved and the exposed skin was sprayed with disinfectant. Endotoxin-free plasmid for injection was prepared in a solution of 0.9% NaCl at a concentration of 500 μg/ml for reporter gene studies or 833 μg/ml for therapeutic studies. DNA (20 μl) was administered by intramuscular (i.m.) injection at three sites, and ultrasound gel (Henleys Medical Supplies Ltd, Welwyn Garden City, Hertfordshire, UK) was then applied to the surface of the skin. Caliper electrodes (384L; BTX Instrument Division, Harvard Apparatus, Holliston, MA, USA) were applied transversely across the quadriceps, and the muscle was electroporated (four pulses at 200 V/cm, duration 20 ms, frequency 2 Hz) using a BTX Electro Square Porator ECM 830 (Harvard Apparatus). The polarity of the electrodes was then reversed and the procedure was repeated.

### Whole body bioluminescent imaging

*In vivo *expression of luciferase was monitored by non-invasive imaging. Mice were given an i.p. injection of 200 μl of luciferin K+ salt (30 mg/ml; Promega Corporation). After 5 minutes, animals were anaesthetised by i.p. injection of 50 μl of a 2:1 mixture of Ketaset^® ^(Fort Dodge Animal Health, Southampton, Hampshire, UK) and Rompun^® ^(Bayer HealthCare, (part of Bayer Schering Pharma AG) Newbury, Berkshire, UK). Anaesthetised mice were then photographed (0.2-second exposure) and imaged for light emission (10 seconds on medium sensitivity for mice treated with pcLuc+ or 5 minutes on high sensitivity for mice treated with pGTLMIK) with the IVIS^® ^100 series (Xenogen Corporation, Hopkinton, MA, USA). Luciferase images were overlaid on the photograph, and emission of light was quantified as photons per steradian per square centimetre using Living Image^® ^software version 2.5.50.1 (Xenogen Corporation) from a defined region of interest around the quadriceps muscles and from control areas of the same size on non-injected quadriceps and abdomen of the same mouse.

### Induction of collagen-induced arthritis

DBA/1 mice (10 to 12 weeks old) were administered Hypnorm™ (0.1 ml, i.p.) and were shaved at the base of the tail. Bovine collagen II (CII) was emulsified with complete Freund's adjuvant at a final concentration of 2 mg/ml, and a total of 0.1 ml was injected intradermally at three sites at the base of the tail. Twenty-one days later, a booster (0.1 ml) consisting of CII emulsified with incomplete Freund's adjuvant (2 mg/ml) was injected intradermally across three sites at the tail base. A further 5 days later, animals were given an i.p. injection of lipopolysaccharide (LPS) (40 μg in 0.1 ml of PBS) (*E. coli *Serotype 055:B5; Sigma-Aldrich Company Ltd, Poole, Dorset, UK) to synchronise disease [21]. The development of arthritis was monitored every 2 to 3 days and was given a clinical score based on visual signs of arthritis: 0.25, swelling in a single digit; 0.5, swelling in more than one digit; 1, swelling and erythema of the paw; 2, swelling of the paw and ankle; 3, complete inflammation of the paw (accordingly, the maximum score for each mouse was 12). The thickness of hind paws was measured using POCO 2T calipers (Krœplin Längenmesstechnik, Schlüchtern, Germany). Mice were monitored until 40 days after immunisation, at which time they were terminated.

Two days after injection of LPS (day 28), animals were assessed for development of arthritis. Animals with a clinical score of less than 4 were used in gene therapy experiments and were administered 50 μg of DNA by i.m. injection (60 μl) at three sites and were electroporated using conditions described above. Blood was collected by tail bleed prior to DNA injection, 2 days after injection, and by cardiac puncture at termination.

### Measurement of dTNFR by ELISA

To measure levels of dTNFR, a microtitre plate was coated with 50 μl of a mouse monoclonal anti-human TNFRII (R&D Systems, Inc., Minneapolis, MN, USA) at 4 μg/ml overnight at 4°C. Plates were washed with PBS and then blocked with 200 μl of 2% casein solution in PBS for 1 hour at room temperature. Plates were washed with PBS containing 0.05% Tween 20 (PBS/Tween) prior to incubation of standards (50 μl of human TNFRII [R&D Systems, Inc.] at a concentration of 1 pg/ml to 1 μg/ml diluted in normal mouse serum or complete media) and samples (50 μl of serum or culture medium) for 3 hours at room temperature. Plates were washed extensively with PBS/Tween before incubation with 50 μl of biotinylated goat anti-human TNFRII (R&D Systems, Inc.) at a concentration of 100 ng/ml for 1 hour at room temperature. Signal was detected using the TMB microwell substrate system (Kirkegaard & Perry Laboratories, Inc., Gaithersburg, MD, USA), the reaction was stopped by addition of 4 M sulphuric acid (100 μl), and absorbance measurements were performed at 450 nm using an EL 312e microplate biokinetics reader (BioTek Instruments, Inc., Winooski, VT, USA). The detection limit of this ELISA was 10 pg/ml.

### Measurement of bioactive Dox in sera

Cells (293T) were plated on 96-well plates at 1 × 10^4 ^cells per well. Twenty-four hours later, they were transfected with 50 ng of GTLMIK plasmid per well using the calcium phosphate precipitation method but with no osmotic shock. The next day, medium was replaced with 100 μl of medium containing heat-inactivated serum samples diluted 1:100. Medium used in this assay contained Tet system-approved foetal calf serum (Clontech, Mountain View, CA, USA), which is free from trace amounts of tetracycline or its derivatives. Cells were then lysed in 50 μl of passive lysis buffer, and luciferase was measured in accordance with the assay method described above. From luciferase values obtained for the Dox standard curve (10 pg/ml to 10 ng/ml), levels of Dox in serum samples were calculated by multiplying by the dilution factor of 100.

### Statistical analysis

Descriptive statistics and significant differences between groups were calculated using the Student's *t *tests for two-sample data of unequal variance (Microsoft^® ^Excel 98 software; Microsoft Corporation, Redmond, WA, USA).

## Results

### Comparison of regulated luciferase expression from pGTLMIK and pGTRTL

**Figure 2:**
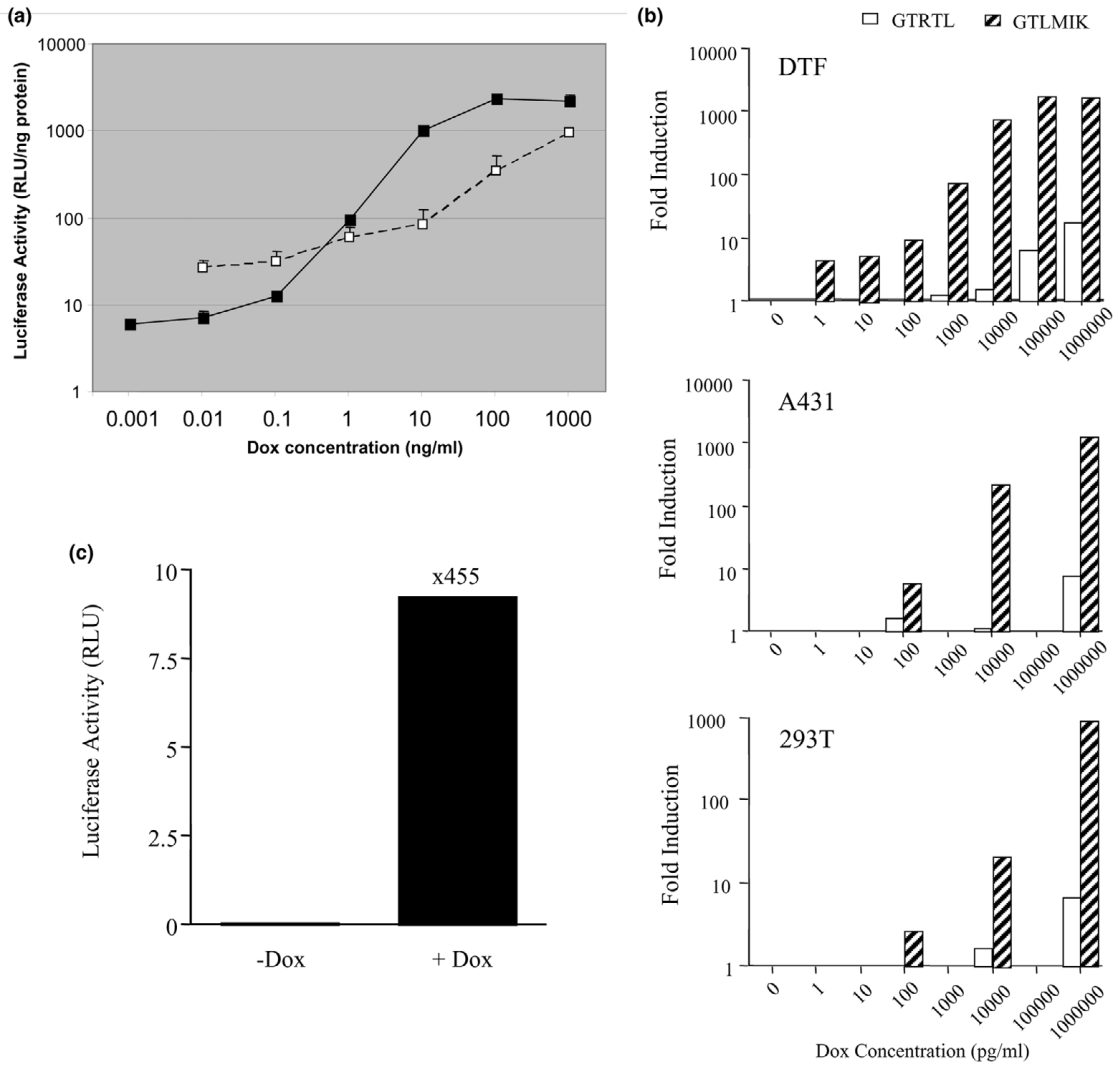
Regulated luciferase expression from self-contained vectors in transfected cells. **(a) **Comparison of regulated luciferase expression from pGTRTL (2 μg) (white squares) and pGTLMIK (2 μg) (black squares) in transiently transfected DBA/1 embryonic fibroblasts with the temperature-sensitive large T antigen (DTF) cells (2 × 10^5 ^cells per well). Cells were grown in doxycycline (Dox)-free or Dox-containing media (concentration range of 1 pg/ml to 1 μg/ml). After 24 hours, luciferase activity was measured in cell lysates. **(b) **Fold induction of luciferase expression from pGTRTL (white bars) and pGTLMIK in response to Dox was determined in DTF, A431, and 293T transiently transfected cells. Cells were transfected as in **(a)**, and fold induction was calculated by dividing the Dox-induced value by the non-induced value for each transfection. **(c) **Regulated expression of luciferase from pGTLMIK (1 μg) transfected into the mouse myoblast cell line C2C12 (1 × 10^6 ^cells) using the amaxa system. Transfected cells were split between the wells of a six-well plate and were either non-induced or induced with Dox (1 μg/ml) for 24 hours. In all panels, luciferase measurements were standardised for protein content and each mean value or calculated value was obtained from triplicate transfections. Vertical lines in **(a) **and **(c) **represent standard error of the mean. RLU, relative light units.

During the construction of pGTLMIK (Figure 1), the function of the intermediate vector pGMIK which expressed both rtTA2S-M2 and *tet*R-KRAB was confirmed by *in vitro *co-transfection experiments (data not shown). Regulated expression of luciferase from pGTLMIK and pGTRTL was compared in a variety of cell lines. Basal expression from GTLMIK in transfected DTFs (1.36 ± 0.36 RLU/ng protein) was significantly (*p *< 0.05) less than basal expression from pGTRTL (55.44 ± 14.5 RLU/ng protein) but was above the non-transfected background (0.06 ± 0.007 RLU/ng protein). Induction of luciferase expression from pGTLMIK in DTFs was sensitive to the lowest Dox concentration (1 pg/ml), whereas GTRTL required a higher Dox concentration (1 ng/ml) to induce expression above basal levels. Maximal levels of expression from pGTLMIK were achieved with 100 ng/ml and 1 μg/ml Dox, whereas pGTRTL required a concentration of 1 μg/ml for full induction, and expression levels remained below the maximum observed with pGTLMIK (Figure 2a). The greater fold induction of luciferase expression from pGTLMIK compared to pGTRTL (Figure 2b) was due to the combination of lower basal and higher induced levels of expression with this vector.

The improved regulated expression of luciferase from pGTLMIK observed in DTFs was also observed in other cells; the vector was responsive to lower Dox concentrations, displaying lower basal expression and demonstrating higher induced expression compared with pGTRTL. Regulated expression of luciferase from GTLMIK was approximately 1,000-fold in mouse fibroblasts (DTF) and human epithelial cells (A431 and 293T) (Figure 2b).

The intended *in vivo *application of the regulated plasmid was transfection of skeletal muscle and so it was important to examine regulated expression from the vector in the myoblast cell line C2C12. Again, efficient regulated expression of luciferase was demonstrated 24 and 48 hours after induction with Dox (1 μg/ml), and the magnitude of induction was approximately 500-fold at both time points (Figure 2c).

### *In vivo *expression of luciferase detected by non-invasive imaging

**Figure 3:**
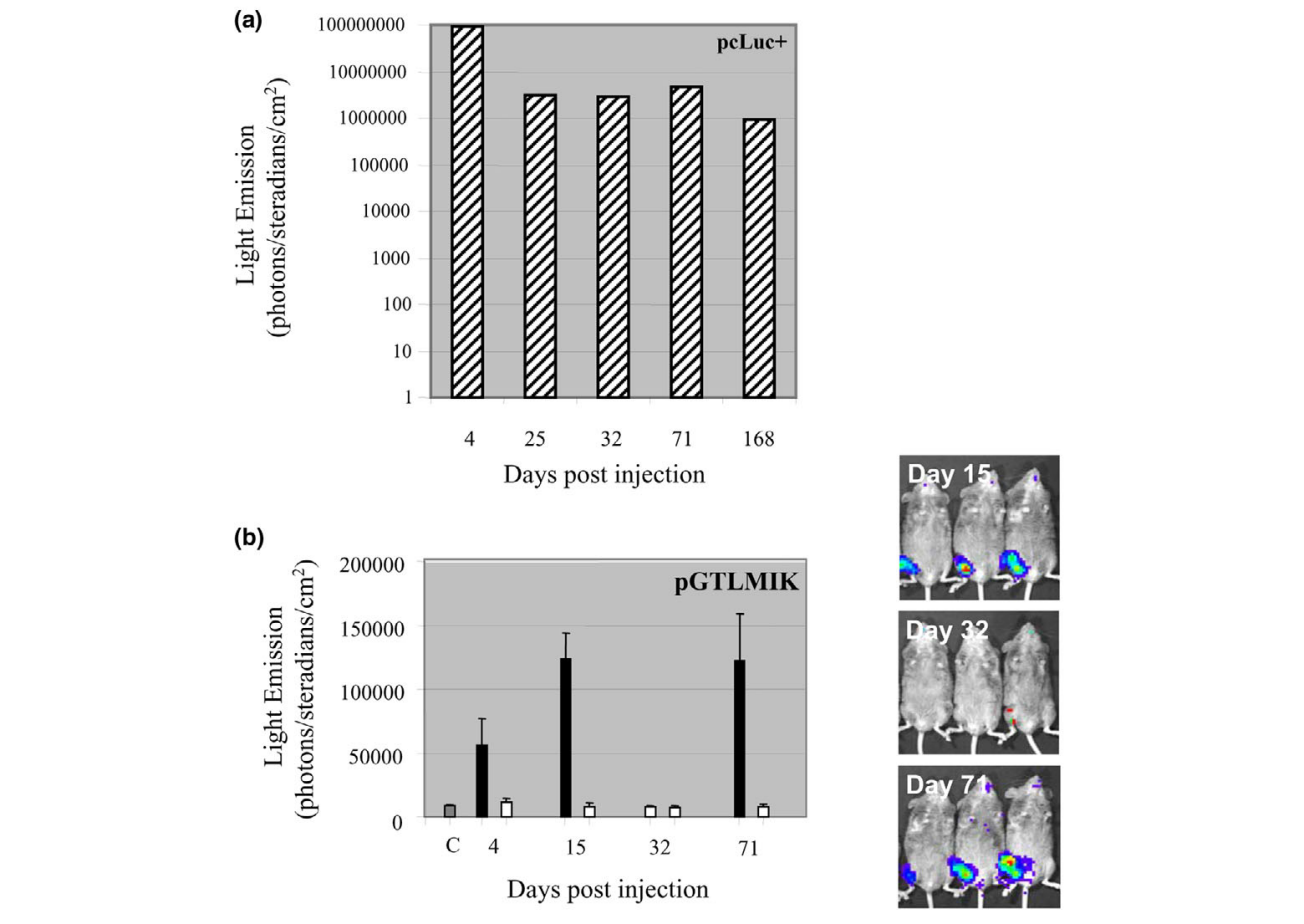
Constitutive and regulated expression of luciferase *in vivo*. Plasmid DNA (30 μg) pcLuc+ **(a) **or pGTLMIK **(b) **was delivered by intramuscular injection with electroporation on day 0. Mice were imaged using the IVIS system on days 4, 15, 32, 71, and 168 (for the pcLuc+-treated mouse) after injection of luciferin substrate and anaesthetisation. Light emission (photons per steradian per square centimetre) was measured from the right leg of DNA-injected mice. For pGTLMIK-treated mice, luciferase expression (black bars) is compared with the left leg (white bars) and with a control (C) region of the same size on the abdomen (grey bar). Mice received doxycycline (Dox) (200 μg/ml) in sweetened drinking water for 15 days after DNA delivery. Dox was then removed until day 67, when the same concentration Dox drink was again supplied. Values for pcLuc+ are obtained from a single mouse, and for pGTLMIK the values are the mean from three mice; vertical lines represent the standard error of the mean. Images obtained from the three pGTLMIK-injected mice on days 15, 32, and 71 are shown on the right. These results confirm that luciferase expression from pGTLMIK can be very efficiently switched on (days 15 and 71) and off (day 32) by addition or removal of Dox from the diet.

The vector pcLuc+ encoding the improved luciferase gene constitutively from a cytomegalovirus (CMV) promoter was used *in vivo *to confirm the transfection procedure to muscle and to determine whether expression levels were maintained for the duration of the experiment. Expression of luciferase from this vector, which was detected on day 4, confirmed that the delivery method was efficient for plasmid DNA (Figure 3a). In addition, i.m. expression levels of luciferase detected by imaging were maintained for at least 5 months (Figure 3a), indicating that plasmid persists long-term in transfected cells and that the CMV promoter is not silenced and confirming that luciferase is non-immunogenic in DBA/1 mice.

Regulated expression of luciferase from pGTLMIK *in vivo *was also examined by bioluminescence imaging. Three treated mice were monitored for expression of luciferase following addition of Dox (200 μg/ml) (days 4, 15, and 71) and removal of Dox (day 32) from drinking water (Figure 3b). These data illustrate that the regulated vector operates *in vivo*, that the tetracycline components of regulation are also non-immunogenic (given that they are expressed long-term), and that the tetracycline promoter P*tet *is not inactivated over the 71-day period of the experiment. Two of these mice were again imaged at 6 months and regulated expression of luciferase was observed (data not shown). The imaging experiments as a whole also demonstrate that detectable levels of luciferase are observed at the site of plasmid injection only and not at distant sites.

### Expression of dTNFR from pGTTMIK in Cos 7 cells

**Figure 4:**
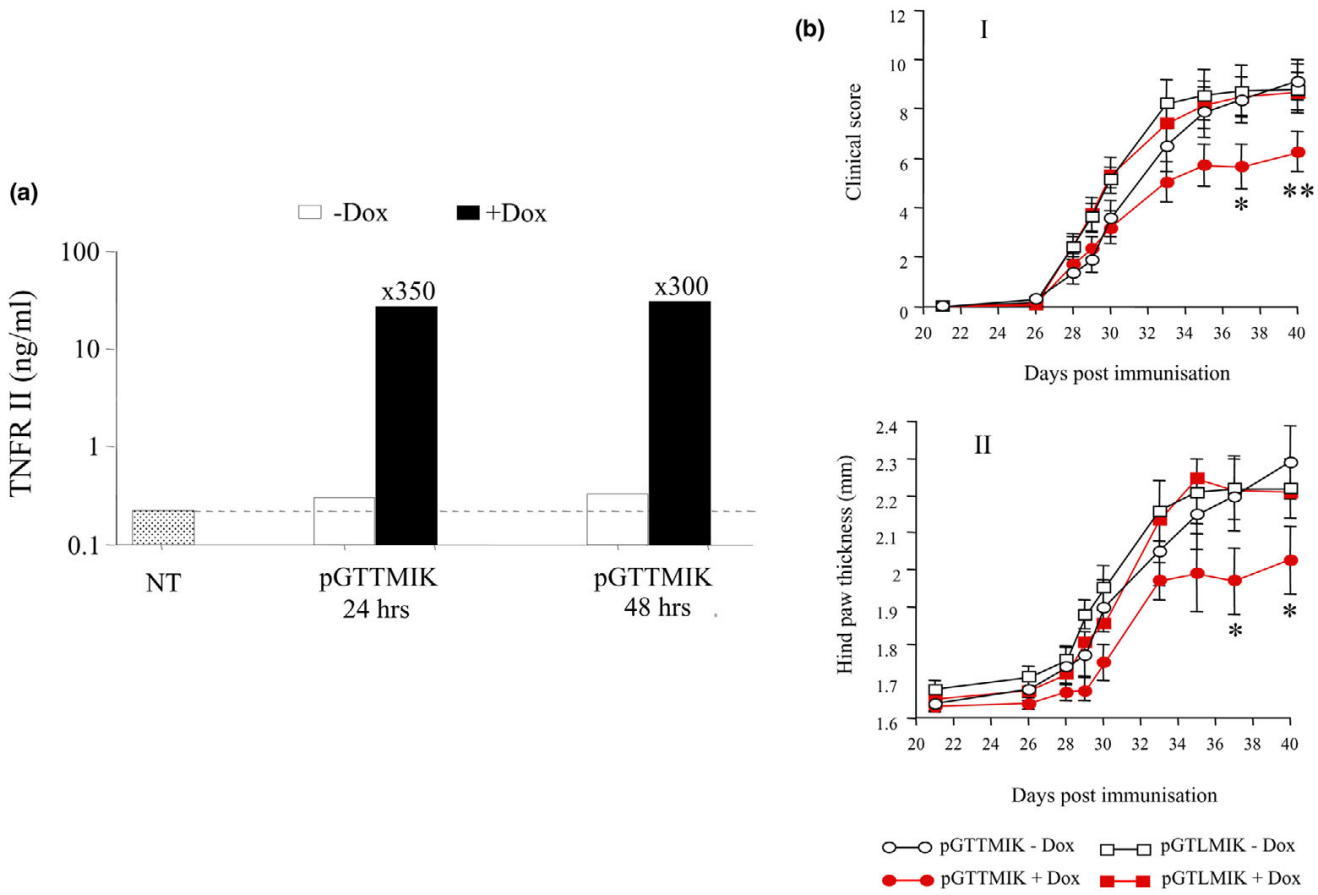
Expression of dimeric tumour necrosis factor receptor II (dTNFR) from pGTTMIK and gene therapy application in the collagen-induced arthritis (CIA) model. **(a) **Cos 7 cells were transiently transfected with 2 μg of pGTTMIK by the calcium phosphate method. Expression of dTNFR was induced for 24 or 48 hours with doxycycline (Dox) (1 μg/ml). Values are the mean of triplicate transfections, and induction values above the black bars allow for the endogenous production of TNFR II from non-transfected (NT) cells indicated by the dotted line. **(b) **Inhibition of CIA in mice treated with pGTTMIK and administered Dox. Progression of CIA both in terms of clinical score (I) and hind paw swelling (II) was inhibited by delivery of pGTTMIK (50 μg) intramuscularly with electroporation on day 28 after immunisation in mice with early disease (clinical score of less than 4). Inhibition was evident in those mice that received Dox in sweetened drinking water. Values are the mean, and vertical lines represent the standard error of the mean. Significant difference of *p *< 0.05 and *p *< 0.02 between the GTTMIK groups given Dox-containing and Dox-free sweetened drinking water are indicated by * and **, respectively.

Regulated expression of dTNFR from pGTTMIK was examined in Cos7 cells. Regulated expression with these cells was approximately 300-fold, and levels of induced expression were similar to those achieved with the vector pGTRTT in a previous study [19] (Figure 4a).

### Inhibition of collagen-induced arthritis by pGTTMIK

Development of arthritis in the collagen-induced arthritis (CIA) model was monitored by measurement of paw thickness and clinical score. In gene therapy experiments, pGTLMIK or pGTTMIK was administered to mice with early arthritis (clinical score of less than 4). Groups of mice then received Dox (200 μg/ml) containing sweetened (10% sucrose) water or sweetened water alone (GTLMIK without Dox [*n *= 13], GTLMIK with Dox [*n *= 12], GTTMIK without Dox [*n *= 11], GTTMIK with Dox [*n *= 10]). Mice that received pGTTMIK and Dox developed significantly lower disease in terms of clinical score and hind paw swelling at days 37 and 40 compared with mice that received pGTTMIK without Dox (Figure 4b).

### Analysis of sera from arthritic mice

**Figure 5:**
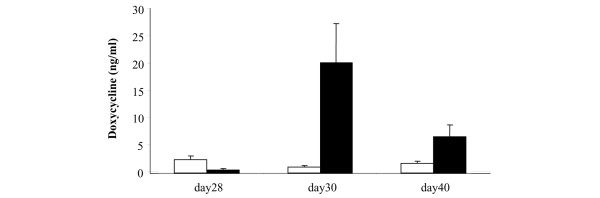
Levels of doxycycline (Dox) in the serum of pGTTMIK-treated mice. Levels of Dox in the serum from mice that were administered sweetened water only (white bars) or Dox (200 μg/ml) (black bars) were determined using pGTLMIK transiently transfected 293T cells. The experiment was performed as decribed in Materials and methods. Dox concentration in the blood was determined from a standard curve of luciferase activity obtained with serum spiked with known Dox concentrations. Mean values are shown, and vertical lines represent the standard error of the mean.

Blood samples were collected before and 2 and 13 days after plasmid DNA (pGTTMIK) administration and were monitored for levels of Dox and dTNFR. Dox levels were elevated in serum collected 2 and 13 days after administration of Dox (Figure 5). Levels were highest after 2 days (20.2 ng/ml) and were lower (6.7 ng/ml) at the end of the experiment. This reduction indicates decreased intake of Dox. A reason for this may be that consumption of Dox-containing water is reduced with progression of arthritis.

**Figure 6:**
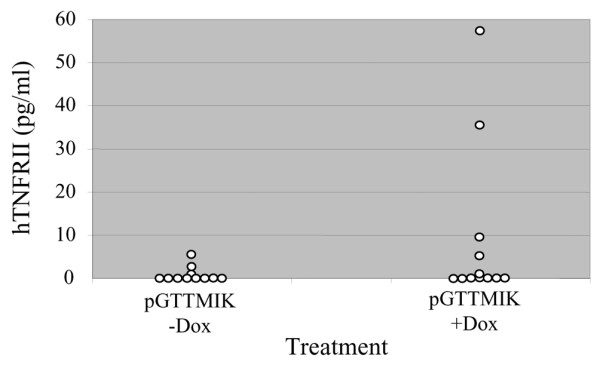
Levels of dimeric tumour necrosis factor receptor II (dTNFR) in the circulation. Enzyme-linked immunosorbent assay (ELISA) was used to measure dTNFR levels in serum samples collected from mice treated with pGTTMIK at the end of the experiment (day 40). Individual samples are illustrated for both the doxycycline (Dox)-treated and untreated groups. The detection limit for the ELISA was 10 pg/ml.

The dTNFR molecule is rapidly removed from the circulation, unlike the Fc-based TNF-α inhibitors, which have long half-lives in the serum. The rapid excretion of dTNFR probably accounts for the low levels of dTNFR in blood of treated mice. Two days after Dox treatment was initiated, no dTNFR was detected. At the end of the experiment, detectable levels by ELISA were determined in the sera of only two mice treated with pGTTMIK and Dox (Figure 6).

## Discussion

Anti-TNF-α is now well established as a biological treatment for RA and achieves an ACR improvement of 20% in approximately 65% of patients [22]. More recently, the treatment has proven effective in other inflammatory diseases, including Crohn's disease [23] and asthma [24], and may even have application in cancer treatment [25]. Importantly, the number of reported side effects remains low, and the greatest concern is the re-occurrence of tuberculosis in some cases. Although the effectiveness of these biologics is welcome, their cost still prohibits their widespread application in the UK and other European countries and precludes their use in poorer nations. We believe that the same or similar treatments could be effectively delivered by a safe gene therapy using plasmid vectors and regulated expression systems.

Plasmids have the advantage that they are non-integrating, cheap to produce, and can give long-term expression when delivered to non-dividing cells. Any gene therapy for a chronic non-fatal disease such as arthritis is required to be safe. Using a method to regulate expression of the transgene will improve safety because expression levels can be adjusted to individual patient needs and if adverse effects occur, expression can be switched off.

We have previously constructed a self-contained plasmid in an auto-regulated format which achieved regulated expression in cells and *in vivo *after i.m. injection with electroporation [11,19]. However, regulation was approximately 50-fold, and basal promoter activity was evident. In this study, we have used the same plasmid backbone from pGL2-Basic vector, and an important feature of this vector is the SV40 polyadenylation signal, which is bidirectional and can therefore reduce the vector size by terminating expression of two genes. In the improved vector format, expression is not auto-regulated, because the constitutively produced *tet*R-KRAB actively represses P*tet *activity in the absence of Dox. Repression by the KRAB domain is achieved by binding of Kap1 and consequent formation of a scaffold to recruit heterochromatin protein 1, histone deacetylases, and Setdb1, leading to chromatin condensation [26]. This repression can act over long distances and has been shown to repress a CMV promoter up to 3 kilobases from the site of KRAB binding [27]. For this reason, the non-encoding *Zeo *gene and downstream polyadenylation signal (500 base pairs) are included in the vector as a spacer to increase the distance between the P*tet *and the constitutive SV40 promoter to approximately 3.3 kilobases. In this improved regulated vector, we have incorporated an internal ribosome entry site (IRES) for co-expression of rtTA2S-M2 and *tet*R-KRAB which can result in decreased expression of the second gene [28]. However, for the purpose of co-expressing the components for tetracycline-regulated gene expression, others have also successfully used an IRES [14,16].

Efficient regulated expression was achieved in a wide variety of cell lines, including 293T cells in which the tetracycline system has previously been shown to function poorly [29]. The vector also functioned well in the myoblast cell line C2C12, which is an indicator for activity *in vivo *when delivered to skeletal muscle. In all cell lines examined, regulated expression of luciferase from pGTLMIK was in the range of 500- to 2,000-fold and expression was regulated more tightly than with pGTRTL. Regulated luciferase expression from pGTLMIK was responsive to Dox concentration from 1 pg/ml to 1,000 ng/ml. The improved gene regulation with pGTLMIK compared to pGTRTL is due to the active repression of the P*tet *by *tet*R-KRAB in the absence of Dox; in the presence of low Dox (from 1 pg/ml), this repression is reversed, and at higher Dox concentrations (from 100 ng/ml), maximal activation of the P*tet *by rtTA2S-M2 occurs in accordance with the original description of this activator [13]. An advantage of expressing both *tet*R-KRAB and rtTA2S-M2 is that the P*tet *is bound in the absence and presence of Dox and this will limit potential interaction of endogenous molecules such as GATA factors with the promoter [30].

Intramuscular injection of plasmid DNA combined with electroporation is an efficient method to transfect skeletal muscle. Constitutive expression of luciferase from pcLuc+ from a CMV promoter was maintained at a consistent level for 5 months, confirming that luciferase is not immunogenic and that the CMV promoter is not silenced in this period *in vivo*. Methylation of long terminal repeat sequences is well established as a mechanism for the silencing expression of retroviral integrated transgene expression [31]. Rapid methylation of the CMV promoter incorporated in an adenoviral vector was also reported *in vivo *following i.m. virus delivery [32]. By contrast, the results observed in this study support the idea that when the CMV promoter is incorporated in episomal plasmid DNA, it is not methylated and remains functional [33]. Regulated expression of luciferase from pGTLMIK is also maintained 6 months. This indicates that the SV40 promoter driving expression of the MIK cassette remains active, contrary to a previous report that its function is transient in skeletal muscle [34]. The safety of the vector could potentially be further improved by replacing the ubiquitous SV40 promoter with a muscle-specific promoter such as the MCK (muscle creatine kinase) promoter [35], which would restrict expression from the plasmid to transfected skeletal muscle and preclude expression from plasmid DNA that disseminates from the injection site. Equally, transfection of potential antigen-presenting cells such as skeletal stem cells located within skeletal muscle could lead to immune rejection of presented transgenes [36]. Again, this could be circumvented by selectively targeting expression with a muscle-specific promoter [37].

TNF-α inhibitors are very effective in the treatment of patients with RA, but in the CIA model TNF-α inhibitors slow the progression of disease when delivered after disease onset but do not reverse disease [38,39]. In mice with established CIA, we show that Dox-induced expression of dTNFR from pGTTMIK inhibited the progression of disease. This finding is also consistent with our previous observations that both constitutive expression and Dox-induced expression of dTNFR during the early stage of CIA inhibit disease development [19]. Similarly, gene therapy delivering other TNF inhibitors such as a dimeric TNFRI [40] and TNFRI and TNFRII Fc molecules [40,41] expressed constitutively from plasmid DNA is also effective in the treatment of CIA.

The same hurdles for treatment of CIA and RA exist. DNA must be efficiently delivered, level of gene expression is dependent on Dox intake, and therapeutic effect requires inhibition of TNF-α. In mice, delivery of plasmid DNA to skeletal muscle combined with electroporation is effective, but when the expressed transgene is not readily detected, transfection cannot be confirmed. It would be preferable to co-express a reporter gene whose expression could be used to confirm muscle transfection. In terms of plasmid delivery in primates, direct injection into muscle is less efficient than in rodents [42] and may indicate that alternative delivery methods will be required for treatment of patients. One potential method is intravenous hydrodynamic injection, which is equally effective in rodents and primates and may prove efficient for clinical application [43].

Administration of Dox (200 μg/ml), a tetracycline analogue in drinking water, led to levels of bioactive Dox detected in the blood from day 2 to the end of the experiment. Because Dox was delivered to a cage and not to individual animals, there may have been considerable inter-animal variation in the amount of Dox delivered. Development of arthritis and consequent restriction of movement may also have affected Dox intake. Interestingly, the levels of bioactive Dox detected in sera (approximately 10 ng/ml) are considerably lower than peak blood levels (1 to 2 μg/ml) achieved in patients receiving 100 mg of Dox daily and are also less than 800 ng/ml, which is the minimal effective anti-microbial concentration [44]. This substantial difference may be due to poor uptake of Dox delivered orally in mice. Previous observations with rats and rabbits have shown that tetracycline delivered in drinking water up to concentrations of 4,000 μg/ml achieved low (300 ng/ml) or undetectable tetracycline levels in the sera (limit of assay 200 ng/ml) [45,46]. The low levels of Dox detected in the present study confirm that the tetracycline system operates well at low Dox concentrations, and importantly, these Dox levels are significantly lower than amounts required for anti-microbial activity.

*In vitro *dTNFR is as effective as etanercept-like molecules at inhibiting TNF-α activity [17] and has previously been shown to be therapeutic in both the CIA model [19,47] and a multiple sclerosis model [48]. In the present study, expression of dTNFR was detected in the blood in only two mice, and in previous studies dTNFR has been undetected in the blood [19,48]. It is intriguing how low levels of dTNFR inhibit the progression of CIA when the dTNFR is not readily detected in the blood. The dTNFR molecule is excreted in the urine, the same route as that of endogenous soluble TNFRs [48]. Due to the rapid half-life of the molecule, it is feasible that TNF-α bound to dTNFR will be rapidly eliminated by this route. By contrast, etanercept-like molecules incorporating the immunoglobulin Fc portion have a long half-life in the blood and bound TNF-α is retained in the system and not rapidly cleared [49]. When delivered as protein, a molecule that has a long half-life and therefore reduces the frequency of re-administration is preferred; but for gene therapy, in which a molecule is continuously produced, it may be preferable for the molecule to be rapidly excreted [50].

The investigation reported here builds on our previous research and demonstrates the improved regulated expression achieved with a second generation of self-contained vector. We believe that this type of vector system can be further developed for clinical application. Plasmid vectors have the major advantages that they are non-integrating and non-immunogenic and can therefore be re-delivered safely, which is likely to be a requirement in the treatment of chronic diseases such as RA. By contrast, all viral vectors contain proteins that elicit an immune response that complicates or precludes their re-administration. Before plasmid treatment for chronic disease can progress to the clinic, efficient delivery is required and this may be feasible by hydrodynamic injection [43]. For RA, TNF-α remains an obvious target, but further studies need to determine the inhibitor with the ideal pharmacokinetic profile for gene therapy application.

## Conclusion

Anti-TNF treatment for RA could be delivered safely by gene therapy through the use of a non-integrating vector and the use of an efficient gene regulation system. In this paper, we describe a novel self-contained transcriptionally regulated plasmid vector encoding a TNF inhibitor which fulfills these requirements. This gene therapy is effective in mice, but for application in the clinic, the vector will require additional modification to improve safety and the components for tetracycline gene regulation will need to be engineered to prevent immunogenicity.

## Abbreviations

ACR = American College of Rheumatology; CIA = collagen-induced arthritis; CII = collagen II; CMV = cytomegalovirus; Dox = doxycycline; dTNFR = dimeric tumour necrosis factor receptor II; ECACC = European Collection of Cell Cultures; ELISA = enzyme-linked immunosorbent assay; i.m. = intramuscular; i.p. = intraperitoneal; IRES = internal ribosome entry site; LPS = lipopolysaccharide; PBS = phosphate-buffered saline; P*tet *= tetracycline-responsive promoter; RA = rheumatoid arthritis; RLU = relative light units; *tet*R-KRAB = tetracycline repressor-Kruppel-associated box; TNF-α = tumour necrosis factor-alpha; TNFR = tumour necrosis factor receptor.

## Competing interests

The authors declare that they have no competing interests.

## Authors' contributions

DG was involved in all aspects of the study. NY contributed to plasmid construction. RF was involved in plasmid preparation and execution of *in vivo *studies. MCS participated in cell assays and contributed to drafting the document. YC contributed to planning of experiments and to content of the document. All authors read and approved the final manuscript.
